# On Teething

**Published:** 1851-10

**Authors:** A. Canton


### ARTICLE XV.

*On Teething. From a Treatise on the Teeth.* By Mr. A. Canton.

The process of teething, as the eruption of the temporary and permanent set of teeth is termed, is one of the utmost interest and importance to the medical attendant, the anxious parent, and to the child itself. In very many instances it unfortunately happens, that the absorption of the gum, and dental sac, set up by the onward progress of the advancing tooth, excites great irritation and fever in the animal economy, which not unfrequently settles in some one or other of the more important organs of the body, and induces disease, which, if not speedily combated by the resources of the medical art, may either lead to permanent and irretrievable mischief, or to the absolute destruction of life. It behooves, then, all who are interested in the welfare of children, to watch carefully at this period of their infantile existence for the signs and evidences of commencing irritation in any part of the system, and as soon as they appear, however trifling they may be, more especially if there be heat and swelling of the gums, with the other symptoms of approaching teething, at once to seek for that medical assistance, by which alone, the impending mischief may be warded off.

We have already given a brief sketch of the development of the teeth, during the period the embryo is carried in the mother's womb, and have brought up our remarks to the time prior to dentition. The teeth continue to grow after the birth of the child, until, in the average of instances, the crown in full growth makes its appearance externally, having caused, by the pressure it exerts, the removal of all the parts lying over it. The fang is not complete until after the crown of the tooth has presented itself above the level of the gum. Before this occurs, certain changes take place in the gums, which indicate to the experienced eye and finger, that nature is urging forward the eruption of a tooth. The gum becomes broader, swollen, hot, and tender; the child is constantly dribbling, and is never easy unless it has a finger, or some substance in its mouth, to press upon the inflamed gum. Much pressure, however, causes a fit of crying, and the little sufferer frequently, in addition, presents signs of general irritation and irritability. It is fretful, frequently crying, and is pacified with difficulty; the cheeks are flushed, sleep restless and disturbed, and the stomach and bowels are more or less disorded. In some instances, however, the teeth are cut with little or no disturbance of the health, and almost the first indication given that the process of dentition has been going on, is the appearance of the tooth above the level of the gum.

The first teeth of the temporary set are cut in general about the sixth, seventh, or eighth month of infantile life; these are the central incisors of the lower jaw, and they are followed in the course of a week or two by the same teeth in the upper jaw. It should be fully understood, however, that in stating the sixth, seventh, or eighth month, as the period when these teeth are first seen in the mouth, the average is taken of the process of teething in healthy children. Teeth have been cut much earlier, and again the process has been delayed till the tenth, and even the twelfth month, the time of their appearance varying greatly in different children, owing to the state of their health, their freedom from disease, hereditary or acquired, the strength of their constitution, &c. Again some children cut their teeth across, as it is termed, that is to say, certain teeth appear above the gum in an order different to that usually met with; the canine perhaps before the incisors, and certain of the molar teeth are found to be making their way most irregularly.

In the usual course of nature, however, the central incisors of the lower jaw are cut about the time already stated, and their appearance is followed some days afterwards by that of the corresponding teeth in the upper jaw. The lateral incisors of the upper jaw next make their way; this occurs about the tenth or eleventh month, and those of the lower jaw soon follow. A month or two after these, we have the anterior molars of the lower jaw piercing through the gum, and soon after those of the superior maxilla. The canines next appear, and lastly the second milk molars, between the age of two years and two and a half.

The irregularities so often encountered among the permanent teeth, are rarely met with in the temporary, either as regards position, number, form, or date of eruption. The well known, but somewhat apocryphal tale respecting Richard III, has, however, had its parallel of late date. Tomes mentions a child, five weeks old, whose mother complained that itwas born with two teeth in the lower jaw, by which her breast, and its own upper jaw, had been injured. On examination, he found two sharp-edged, rough-surfaced incisor teeth sticking up from the center of the lower jaw. They were ill-shaped, imperfectly coated with enamel, and loose in the gum, and stood across, instead of in a line with the alveolar arch. These were removed, and it was found that the fangs were not more than one-third developed. In fact, the teeth had attained about the normal amount of development for the age of the child, but had been protruded through the gums before they were fitted for eruption. An after-process had been effected before the preparatory one had been completed. A similar case has occurred in my own practice.

Dr. Brown, an American physician, mentions a still more extraordinary example, which he published in the *American Journal of Dental Surgery*. A child was born with the central incisors through the gums. They were extracted. Two other children were afterwards born of the same mother, in each of whom the same singular anomaly was found. Their teeth were allowed to remain. In each case the children were females. Dr. Crump, another American physician, reports a case of full dentition, at birth, in a still-born negro child. The sockets were very imperfectly formed. Dr. Lethbridge, it is said, has met with a similar instance.

Imperfections in dentition are of occasional occurrence. Sometimes the central incisors are absent altogether; in others again, even when the child has attained its sixth year, it may have a few front teeth only. Two cases have been recorded in which the persons reached old age, without even cutting a tooth. In a case exhibited by Dr. Brinton at the Pathological Society, the alveolar processes of the upper jaw-bones were wanting, but the canine teeth were present on each side, lying parallel to the maxilla. I myself examined a child, whose mouth was apparently well formed. Nevertheless I detected the absence of the right canine tooth of the lower jaw during the first dentition. This was a deficiency that a dentist or a surgeon only could detect, as the jaw appeared to possess its full complement of teeth, there not being any vacancy in the place where the canine should have been found. As I looked upon this as an interesting specimen of dental pathology, I made a model of the mouth, which is now in the possession of my friend, Mr. Tomes.

It has been already stated that the irritation caused by the process of dentition will, in many children, induce disorder, and even permanent disease of other, and important, organs of the body. The first signs indicative of the commencement of that process are mostly attended by fever, and general disorder of the system. The child is irritable and fretful; the skin hot and dry; the bowels disordered; the scalp hot; the eyes heavy and dull, or preternaturally bright; the mouth slavers, the gums are swollen, broader, and more red than usual, hot and painful to the touch: the appetite is lost; the little patient taking the breast by fits and starts, and speedily losing its hold of the nipple. The bowels are generally disordered, the stools being relaxed, and often of a pale, pasty appearance, or green, or clay-colored. Frequent purging, with griping pain, is not an uncommon concomitant of dentition. The head, however, may be the part that sympathises most during teething; when the irritation has been continued for some little time, or even very early during the attack, the infant may be seized with convulsive fits, recurring often with great rapidity, and ending not unfrequently in death, if the appropriate preventive, and remedial measures, be not adopted. A less severe form of disorder, thus induced, may, however, attend on this cause of irritation; eruptions on the scalp, cutaneous inflammations, and discharge from behind the ears, inflammation of the eyes, &c., all in childhood owe their origin at times to the concomitant disorder of dentition. Water on the brain and epilepsy, generally of a temporary, but sometimes of a permanent character, may be thus excited. Again, palsy, affecting one or more limbs, may be induced, and may become permanent, or even prove the precursor to a fatal termination.

On this point, Dr. Henry Davies, late of Saville-row, Physician to the British Lying-in Hospital, and now of Brighton, has added his testimony in proof of the very serious diseases which are sometimes set up in consequence of dentition. In a communication which he addressed to the Medical and Chirurgical Society, he describes the case of a female infant, rather more than a year old, who had cut two lower teeth well; but who one day, having been previously very lively, and having taken her usual exercise in the baby-jumper, and been put to bed apparently well, soon after became restless, and slightly shivering. Early the next morning, she uttered a peculiar cry, and the nurse gave her some Dalby's carminative. This not affording any relief, she was put into a warm bath, and had a dose of castor oil. A few hours after, she was violently sick, turned cold, and looked blue about the mouth, nose, and eyes. She was then put into a warm bath again, when she appeared to lose the entire use of her limbs, and her countenance became vacant. Dr. Davies did not see her till the evening of the second day—much valuable time being thus lost. She was perfectly conscious; head cool, and the temperature of the surface generally natural, the pupils contracting under the influence of light: pulse moderately frequent, small and languid: tongue slightly coated. The upper extremities were devoid of all power of motion, or sensation, even from pinching or pricking with a pin; but the lower extremities were drawn up, on her feet being tickled. Nothing unnatural could be detected in the spine or elsewhere. Leeches had been applied behind the ears; a blister was placed between the shoulders, and aperients, &c., were administered. During the five weeks that the child lived, no change took place in the paralytic symptoms, and she remained conscious to within a few hours of death. During this time she cut six teeth. The appearances after death showed some degree of determination of blood to the brain, which, with the upper part of the spinal marrow, was peculiarly firm. There was an ounce and a half of sanguineous fluid covering the surface of the brain.

In commenting on this very interesting case, Dr. Davies stated that palsy, coming on suddenly, not preceded nor accompanied by any apparent disease of the brain, is by no means uncommon in children between the first and tenth years. In all these cases, he believes—and facts fully demonstrate the truth of his opinion—that the predisposing cause is the process of dentition. The exciting, or immediate cause has, he says, in the majority of cases, appeared to be some derangement of the digestive organs. In this peculiar case, he remarks that the exciting cause was the succession of shocks received by the spinal cord through the use of the baby-jumper.

This interesting and valuable instance of palsy occurring in an infant, bears directly on the statements that have been advanced respecting the dangerous consequences of neglected dentition. Had this poor little sufferer been subjected to the innocuous and highly beneficial operation of lancing the gums on the morning she was taken ill, it is more than probable that all the serious symptoms which ensued would have been prevented, and the infant still alive to rejoice the hearts of its parents. That the distress and nervous derangement dependent on dentition were the predisposing cause of the palsy, we have the authority of a distinguished physician, whose energies and medical knowledge have been especially directed to the investigation and treatment of the diseases of women and children, for believing. Every fact in physiology and pathology at all bearing on the subject confirms this opinion. The cases yet to be narrated, and the facts which have been already made known, are such as not to admit of any doubt upon the subject. Warm baths and aperients, and the application of leeches, were relied on to remove a disease affecting the frame generally, and the nervous system especially, while the simple but most efficient remedy, lancing the gums for the removal of the local cause of this general mischief, was apparently wholly neglected; at all events, no mention is made of its performance in the records of the case in the medical journals; it is therefore only fair to presume that it was not had recourse to. In this respect no blame attaches to the medical men, as their assistance apparently was not sought until the domestic pharmacopœia had been exhausted, and the disease was fully established. It is much to be regretted that the non-performance of this trifling operation led to the sacrifice of a human life, but it is to be hoped that the record of the painful results following its neglect will serve to open the eyes of parents and nurses to the folly of which they are guilty, in offering a sustained and almost systematic opposition to lancing the gums—a proceeding which has been the means of preventing, and of removing a vast amount of infantile disease, and consequently of saving many lives.

Similar testimony is borne by Dr. Marshall Hall, in his work, on the Diseases and Derangements of the Nervous System, in which he has published the case of a little girl, twenty months old, a daughter of Dr. Grant, of Thayer-street, who, while suffering from dentition, lost the power of elevating the right arm—that of closing the hand and of bending the forearm remaining. There was not any symptom of brain affection present. The case was clearly one of palsy from teething. The gums over the four eye-teeth, which were all coming forward, consequently were freely lanced every second day; the bowels well moved, and the diet strictly regulated. For fear of hidden disease within the head, two leeches were applied. A few days after the attack of palsy, the little girl was seized in the early part of the night, with a fit of crowing inspiration. This confirmed Dr. M. Hall in his opinion as to the cause of the attack, and the event justified the view he had taken. The child recovered perfectly, without any energetic remedy being used for the cerebral affection, by continued attention to the state of the gums, the stomach, and the bowels: an event, he says, which could scarcely have occurred from such simple measures, had there been such decided affection arising from disease of the brain. The palsy and the cough completely disappeared when the four teeth made their appearance in the mouth.

(To be Continued.)

